# Validation of the PI-RADS language: predictive values of PI-RADS lexicon descriptors for detection of prostate cancer

**DOI:** 10.1007/s00330-020-06773-1

**Published:** 2020-03-26

**Authors:** Madhuri M. Rudolph, Alexander D. J. Baur, Matthias Haas, Hannes Cash, Kurt Miller, Samy Mahjoub, Alexander Hartenstein, David Kaufmann, Roman Rotzinger, Chau Hung Lee, Patrick Asbach, Bernd Hamm, Tobias Penzkofer

**Affiliations:** 1 Department of Radiology, Freie Universität Berlin, Humboldt-Universität zu Berlin and Berlin Institute of Health, Charité–Universitätsmedizin Berlin, Augustenburger Platz 1, 13353 Berlin, Germany; 2 Department of Urology, Freie Universität Berlin, Humboldt-Universität zu Berlin and Berlin Institute of Health, Charité–Universitätsmedizin Berlin, Charitéplatz 1, 13353 Berlin, Germany; 3 Department of Urology, Universität zu Köln, Uniklinik Köln, Kerpener Str. 62, 50937 Köln, Germany; 4 Department of Radiology, Tan Tock Seng Hospital, Singapore, Singapore; 5 Berlin Institute of Health (BIH), Anna-Louisa-Karsch-Str. 2, 10178 Berlin, Germany

**Keywords:** Magnetic resonance imaging, Prostatic neoplasms, Predictive value of tests, Biopsy

## Abstract

**Objectives:**

To assess the discriminatory power of lexicon terms used in PI-RADS version 2 to describe MRI features of prostate lesions.

**Methods:**

Four hundred fifty-four patients were included in this retrospective, institutional review board–approved study. Patients received multiparametric (mp) MRI and subsequent prostate biopsy including MRI/transrectal ultrasound fusion biopsy and 10-core systematic biopsy. PI-RADS lexicon terms describing lesion characteristics on mpMRI were assigned to lesions by experienced readers. Positive and negative predictive values (PPV, NPV) of each lexicon term were assessed using biopsy results as a reference standard.

**Results:**

From a total of 501 lesions, clinically significant prostate cancer (csPCa) was present in 175 lesions (34.9%). Terms related to findings of restricted diffusion showed PPVs of up to 52.0%/43.9% and NPV of up to 91.8%/89.7% (peripheral zone or PZ/transition zone or TZ). T2-weighted imaging (T2W)–related terms showed a wide range of predictive values. For PZ lesions, high PPVs were found for “markedly hypointense,” “lenticular,” “lobulated,” and “spiculated” (PPVs between 67.2 and 56.7%). For TZ lesions, high PPVs were found for “water-drop-shaped” and “erased charcoal sign” (78.6% and 61.0%). The terms “encapsulated,” “organized chaos,” and “linear” showed to be good predictors for benignity with distinctively low PPVs between 5.4 and 6.9%. Most T2WI-related terms showed improved predictive values for TZ lesions when combined with DWI-related findings.

**Conclusions:**

Lexicon terms with high discriminatory power were identified (e.g., “markedly hypointense,” “water-drop-shaped,” “organized chaos”). DWI-related terms can be useful for excluding TZ cancer. Combining T2WI- with DWI findings in TZ lesions markedly improved predictive values.

**Key Points:**

*• Lexicon terms describing morphological and functional features of prostate lesions on MRI show a wide range of predictive values for prostate cancer.*

*• Some T2-related terms have favorable PPVs, e.g., “water-drop-shaped” and “organized chaos” while others show less distinctive predictive values. DWI-related terms have noticeable negative predictive values in TZ lesions making DWI feature a useful tool for exclusion of TZ cancer.*

*• Combining DWI- and T2-related lexicon terms for assessment of TZ lesions markedly improves PPVs. Most T2-related lexicon terms showed a significant decrease in PPV when combined with negative findings for “DW hyperintensity.”*

**Electronic supplementary material:**

The online version of this article (10.1007/s00330-020-06773-1) contains supplementary material, which is available to authorized users.

## Introduction

Multiparametric magnetic resonance imaging (mpMRI) has emerged as a vital tool in the diagnosis of prostate cancer (PCa). Along with the widespread adoption of prostate MRI, a standardized interpretation and reporting of mpMRI findings have become necessary [1, 2]. For this purpose, the Prostate Imaging Reporting and Data System (PI-RADS) was developed, based on a synthesis of expert consensus and available evidence. The revised PI-RADS version 2 (v2) was released in December 2014 [3]. Recently, in March 2019, modifications to PIRADS v2 have been published constituting an updated version termed PI-RADS v2.1 [4]. As PI-RADS is intended to be a document in evolution, studies have been encouraged to test its efficacy.

Despite recent developments in improving quantitative radiological methods, the vast majority of prostate cancer diagnosis on MRI is performed in the traditional “radiologist reporting” setting. Thus, the vocabulary and subjective assessments of the radiologist are the cornerstones of the reports’ validity.

PI-RADS scoring is done by assessing lesions’ features on T2-weighted (T2WI), diffusion-weighted (DWI), and dynamic contrast-enhanced imaging (DCE). The assessed criteria include lesions’ signal intensity, shape, margins, size, and invasive behavior/extraprostatic extension. The PI-RADS v2 document provides a lexicon with defined descriptors in its appendix (Appendix III) that constitutes the very foundation of this assessment [3]. Analyzing and understanding this very foundation of PI-RADS could enable us to identify descriptors with high diagnostic accuracy, thus allowing these to be incorporated more prominently into the scoring criteria, while reducing the importance of descriptors with low accuracy.

Lexicon terms and their definitions remain largely unchanged in PI-RADS version 2.1, with the only changes being the redefinition of the term “negative DCE,” a new definition of the term “marked” and the introduction of the terms “typical” and “atypical BPH nodule.”

A number of studies have assessed the diagnostic value of the PI-RADS score [5–9], but to date, only sparse data is available concerning the underlying terminology. To the best of our knowledge, only few studies with small study populations have addressed the discriminatory power of individual lexicon terms [10, 11]. Therefore, the objective of this study is to systematically assess the diagnostic value of individual descriptors as specified in the PI-RADS v2 lexicon in a large patient cohort.

## Materials and methods

### Patients

**Fig. 1:**
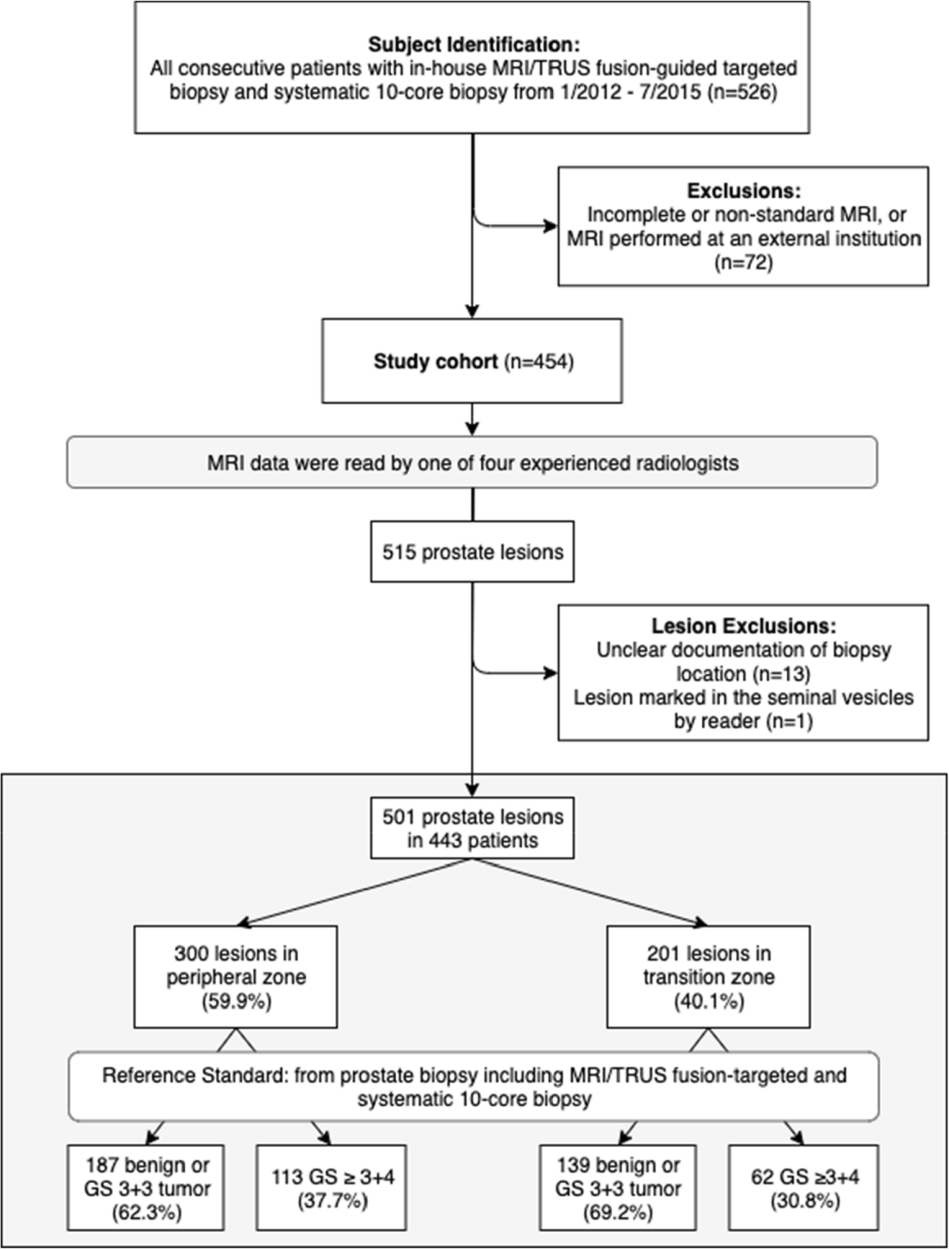
Flow chart with inclusion and exclusion criteria, as well as lesion localization and histopathological outcomes. All men received prostate biopsy including MRI/TRUS fusion-guided targeted biopsy and systematic 10-core biopsy. mpMRI, multiparametric magnetic resonance imaging.; MRI/TRUS, MRI/transrectal ultrasound; GS, Gleason score

The inclusion criteria for this retrospective study were the availability of a prostate MRI between January 2012 and July 2015 and subsequent in-house targeted MRI/TRUS fusion biopsy (TB) in combination with a 10-core systematic biopsy in the same session. From a total of 526 eligible patients, 72 patients with incomplete or non-standard MRI or MRI performed at an external institution were excluded. These exclusions left a final cohort of 454 patients. Patient characteristics are summarized in Table 1. Figure 1 contains a STARD 2015‑compliant patient flow diagram [12] for the study. The study protocol was approved by the institutional review board and patient consent was waived due to the retrospective design of the study. Subgroups of the same collective with various study endpoints have been included in earlier publications pertaining to the accuracy of prostate biopsies [13–18].

**Table 1:** Patient characteristics. Values are given as mean ± standard deviation [range] for continuous variables and absolute frequency (relative frequency) for biopsy results. *PSA*, prostate-specific antigen; *PIN*, prostatic intraepithelial neoplasia

Parameter	Value
Number of patients	454
Age (years)	66.58 ± 7.76 [38–85]
PSA (ng/ml)	13.17 ± 15.49 [0.65–199]
PSA density (ng/ml^2^)	0.26 ± 0.35 [0.02–3.78]
Prostate volume (ml)	61.81 ± 30.82 [6.07–193.63]
Biopsy result (highest grade)	
No cancer nor inflammation	65 (14.3%)
Inflammation (chronic or acute)	86 (18.9%)
PIN	12 (2.6%)
3 + 3	91 (20.0%)
3 + 4	65 (14.3%)
4 + 3	42 (9.3%)
4 + 4	71 (15.6%)
4 + 5	15 (3.3%)
5 + 4	4 (0.9%)
5 + 5	3 (0.7%)

### MR imaging

All imaging was performed on one of two identical 3-T MRI scanners (Skyra, Siemens Healthineers). The following imaging parameters were used in all patients: axial and coronal T2WI with a resolution of 3.0 × 0.47 × 0.47 mm, axial DWI with a resolution of 3 × 1.4 × 1.4 mm with measured *b* values of 0, 50, and 500 and high *b* value (800, 1000, or a calculated *b* value of 1400 s/mm^2^), and additional T1 axial and T2 axial and sagittal imaging of the whole pelvis. In 242 patients (54.6%), DCE imaging was performed additionally with a spatial resolution of 3 × 1.4 × 1.4 mm, a temporal resolution of 5 s, and a 3 ml/s injection flow (Gadobutrol, Gadovist, Bayer Healthcare).

### Imaging review and lexicon term assessment process

**Fig. 2:**
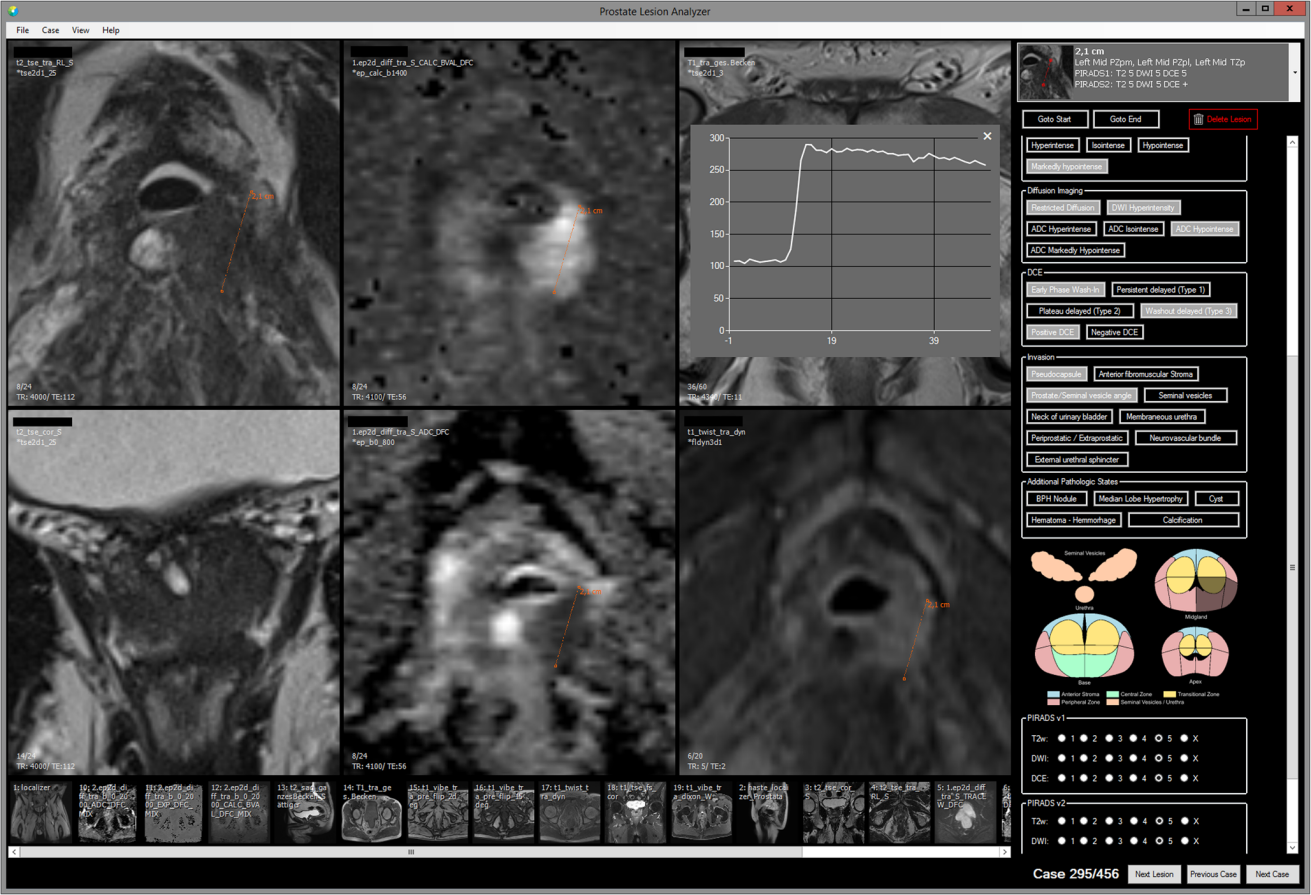
Sample screenshot of the proprietary review software Prostate Lesion Analyzer used in the study. Readers were instructed to mark lesions within the MRI pictures (left, 3 × 2 panels depicting T2WI axial, T2WI coronal, DWI/ADC, T1WI native, and DCE). Matching lexicon terms and lesion localization were selected on the panel on the right side. Definitions of lexicon terms were displayed exactly as specified in the original PI-RADS version 2 document when hovering the cursor over a term. T2WI, T2-weighted imaging; DWI/ADC, diffusion-weighted imaging/apparent diffusion coefficient; T1WI, T1-weighted imaging; DCE, dynamic contrast-enhanced imaging

Four hundred fifty-four MRI imaging datasets were divided into four similarly sized subgroups (113–114 each). Each group was evaluated by one of four readers (A.B., M.H., C.L., P.A.), all board-certified radiologists with more than 5 years of experience in prostate MRI. Each lesion was assessed once by a single reader using a dedicated in-house built reading software. The software presents all imaging in a standardized way to the reader (Fig. 2). Readers were blinded to all patient-related data including the initial radiological report and histopathological results. Readers were instructed to mark the most suspicious lesion or lesions in an MRI and tag every marked lesion with matching lexicon terms complying with the definitions supplied by the PI-RADS v2 lexicon. Definitions of all lexicon terms were displayed in the reading software exactly as specified in the lexicon of the original PI-RADS v2 document [3]. Table 2 contains a full list of the used terms and their classification. All groupings of lexicon terms (DWI-related, shape-related, border-related terms, etc.) were tagged separately. Lesions were attributed to either the peripheral zone (PZ) or the transition zone (TZ) and localized according to the segmentation model used in PI-RADS v2 [3]. Lesions that extended through PZ as well as TZ and lesions that were located in the anterior stroma (AS) or central zone (CZ) were assigned to either the PZ or the TZ group depending on the most probable zone of origin.

**Table 2:** Predictive values of lexicon terms used for lesions in the PZ and TZ. Values are given as PPV in percent (cancer-positive lesions with term marked/all lesions with term marked) and NPV in percent (cancer-negative lesions without term marked/all lesions without term marked). *PZ*, peripheral zone; *TZ*, transition zone; *PPV*, positive predictive value; *NPV*, negative predictive value; *TP*, true positives; *DWI/ADC*, diffusion-weighted imaging/apparent diffusion coefficient; *DCE*, dynamic contrast-enhanced imaging; *DP*, delayed phase

	Terms	Peripheral zone		Transition zone	
		PPV in %	NPV in %	PPV in %	NPV in %
DWI/ADC features	Restricted diffusion	50.7% (104/205)	90.5% (86/95)	40.2% (51/127)	85.1% (63/74)
	DW hyperintensity	52.0% (105/202)	91.8% (90/98)	43.9% (54/123)	89.7% (70/78)
	ADC hyperintense	0.0% (0/3)	62.0% (184/297)	0.0% (0/3)	68.7% (136/198)
	ADC isointense	12.8% (5/39)	58.6% (153/261)	9.8% (4/41)	63.8% (102/160)
	ADC hypointense	42.7% (108/253)	89.4% (42/47)	37.4% (55/147)	87.0% (47/54)
DCE features*	Early phase washin	56.5% (48/85)	75.8% (69/91)	18.4% (7/38)	70.8% (46/65)
	Persistent DP (type 1)	20.0% (10/50)	52.4% (66/126)	26.9% (7/26)	75.3% (58/77)
	Plateau DP (type 2)	38.6% (27/70)	59.4% (63/106)	25.7% (9/35)	75.0% (51/68)
	Washout DP (type 3)	68.2% (30/44)	69.7% (92/132)	25.8% (8/31)	75.0% (54/72)
	Positive DCE	55.4% (56/101)	81.3% (61/75)	28.3% (13/46)	77.2% (44/57)
	Negative DCE	13.6% (8/59)	47.0% (55/117)	26.0% (13/50)	75.5% (40/53)
T2WI features	Hyperintense	15.8% (3/19)	60.9% (171/281)	3.1% (1/32)	63.9% (108/169)
	Isointense	11.1% (1/9)	61.5% (179/291)	5.3% (1/19)	66.5% (121/182)
	Hypointense	34.9% (91/261)	43.6% (17/39)	29.8% (51/171)	63.3% (19/30)
	Markedly hypointense	67.2% (43/64)	70.3% (166/236)	52.5% (21/40)	74.5% (120/161)
Border	Circumscribed	41.0% (55/134)	65.1% (108/166)	20.4% (22/108)	57.0% (53/93)
	Non-circumscribed	25.9% (14/54)	59.8% (147/246)	53.3% (16/30)	73.1% (125/171)
	Indistinct	28.7% (37/129)	55.6% (95/171)	37.7% (26/69)	72.7% (96/132)
	Obscured	37.6% (35/93)	62.3% (129/207)	32.8% (21/64)	70.1% (96/137)
	Irregular	43.8% (46/105)	65.6% (128/195)	50.0% (30/60)	77.3% (109/141)
	Spiculated	56.7% (17/30)	64.4% (174/270)	57.1% (8/14)	71.1% (133/187)
	Encapsulated	11.1% (1/9)	61.5% (179/291)	6.9% (4/58)	59.4% (85/143)
	Organized chaos	14.3% (1/7)	61.8% (181/293)	5.4% (3/56)	59.3% (86/145)
	Erased charcoal sign	80.0% (8/10)	63.8% (185/290)	61.0% (36/59)	81.7% (116/142)
Shape	Round	25.9% (14/54)	59.8% (147/246)	21.4% (9/42)	66.7% (106/159)
	Oval	45.7% (43/94)	66.0% (136/206)	15.6% (12/77)	59.7% (74/124)
	Lenticular	58.6% (17/29)	64.6% (175/271)	44.7% (17/38)	72.4% (118/163)
	Lobulated	56.8% (25/44)	65.6% (168/256)	46.3% (19/41)	73.1% (117/160)
	Water-drop-shaped	100.0% (6/6)	63.6% (187/294)	78.6% (11/14)	72.7% (136/187)
	Wedge-shaped	20.0% (13/65)	57.4% (135/235)	50.0% (1/2)	69.3% (138/199)
	Linear	6.3% (1/16)	60.6% (172/284)	- (0/0)	69.2% (139/201)
	Irregular	38.7% (43/111)	63.0% (119/189)	39.3% (24/61)	72.9% (102/140)
Invasion	Invasion	76.4% (42/55)	71.0% (174/245)	58.9% (33/56)	80.0% (116/145)

*Only for patients with DCE imaging

### Reference standard

Prostate biopsies taken ahead of this study were performed by experienced urologists or interventional radiologists using one of two biopsy devices (Aplio 500, Toshiba or HI VISION Preirus, Hitachi Medical Systems) and consisted of TB and systematic 10-core biopsy. These were used as a reference standard. Histopathological findings were classified according to the Gleason grading system [19]. A Gleason score (GS) of 3 + 4 or higher on TB or in a matching segment on systematic biopsy was considered a positive finding for clinically significant prostate cancer (csPCa). Tumor size or volume was not taken into consideration since size analysis was outside the scope of this study. Histopathological findings that indicated no cancerous changes (no tumor cells, acute prostatitis, chronic prostatitis, prostatic intraepithelial neoplasia, or benign prostatic hyperplasia) and GS 3 + 3 tumor were considered non-csPCA.

### Statistical evaluation

Positive and negative predictive values (PPVs, NPVs) as well as sensitivity and specificity for detection of csPCa were computed for each of the terms within each zone. For TZ lesions, PPVs of shape and border terms in combination with DWI/ADC terms were additionally analyzed. PPVs of term combinations were compared with PPVs of single terms using the generalized score test by Leisenring, Alonzo, and Pepe [20]. Results were declared to be significant if *p* < 0.05. Statistical evaluation was performed using R version 1.1.419 (www.r-project.org) and Microsoft Excel version 16.16.17.

## Results

### Lesion characteristics

The readers marked 515 MRI lesions in the 454 MRI datasets. In 5 patients, no lesions were marked by the readers. Thirteen lesions were excluded due to unclear documentation of biopsy locations and one lesion was excluded due to its location in the seminal vesicles. This left a total of 501 lesions in 443 patients. CsPCa was detected in 175 (34.9%) of the lesions. As shown in Fig. 1, 300 (59.9%) lesions were found in the PZ; prevalence of csPCa in PZ lesions was 113 (37.7%). Two hundred one lesions (40.1%) were located in the TZ; prevalence of csPCa in TZ lesions was 62 (30.8%).

### Diagnostic performance of lexicon terms for PZ lesions

**Fig. 3:**
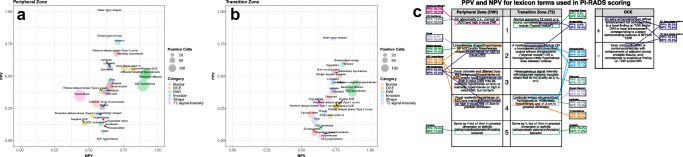
Predictive values of PI-RADS v2 lexicon terms for peripheral zone (**a**) and transition zone (**b**). Current PI-RADS v2.1 assessment criteria with their respective predictive values as found in this study are shown in (**c**). PPV and NPV approximating 1 were considered favorable for terms indicating malignancy. PPV and NPV approximating 0 were considered favorable for terms indicating benignity. PPV, positive predictive value; NPV, negative predictive value; DCE, dynamic contrast-enhanced imaging; DWI, diffusion-weighted imaging

Table 2 and Fig. 3a show PPVs and NPVs of the analyzed lexicon terms. Sensitivity and specificity are presented in Table 4 in the supplementary material. Lexicon terms with the highest PPVs were the following: restricted diffusion (50.7% [104/205]), DW hyperintensity (52.0% [105/202]), early phase washin (56.5% [48/85]), washout delayed phase (68.2% [30/44]), positive DCE (55.4% [56/101]), T2W markedly hypointense (67.2% [43/64]), spiculated (56.7% [17/30]), lenticular (58.6% [17/29]), lobulated (56.8% [25/44]), water-drop-shaped (100.0% [6/6]), and invasion (76.4% [42/55]). Lexicon terms with the highest NPVs were the following: restricted diffusion (90.5% [86/95]), DW hyperintensity (91.8% [90/98]), and ADC hypointense (89.4% [42/47]). Terms indicating benignity showed low PPVs for detection of csPCa, with the term “linear” displaying the lowest PPV of 6.3% (1/16).

In a few cases, the readers also tagged PZ lesions with lexicon terms that are designed to describe TZ lesions (e.g., “erased charcoal sign” and “organized chaos”); these results are given in Table 2 and supplementary Table 4 for the sake of completeness.

### Diagnostic performance of lexicon terms for TZ lesions

Table 2 and Fig. 3b show predictive values of lexicon terms used for TZ lesions. The highest PPVs were found for the following lexicon terms: T2W markedly hypointense (52.5% [21/40]), non-circumscribed (53.3% [16/30]), irregular border (50.0% [30/60]), spiculated (57.1% [8/14]), erased charcoal sign (61.0% [36/59]), water-drop-shaped (78.6% [11/14]), and invasion (58.9% [33/56]). Lexicon terms with the highest NPVs were the following: restricted diffusion (85.1% [63/74]), DW hyperintensity (89.7% [70/78]), and ADC hypointense (87.0% [47/54]). Terms with the lowest PPVs were the following: T2W hyperintense (3.1% [1/32]), T2W isointense (5.3% [1/19]), encapsulated (6.9% [4/58]), and organized chaos (5.4% [3/56]). Figure 3c summarizes predictive values of the most relevant lexicon terms that are currently used as assessment criteria in PI-RADS v2.1.

### PPVs of border and shape terms combined with diffusion-related terms used for TZ lesions

Combining shape and border terms with the term “diffusion-weighted hyperintensity” yielded the most distinctive PPVs for TZ lesions. PPVs of these term combinations are shown in Table 3. In most cases, combining a positive finding of “DW hyperintensity” with a shape- or border-related term increased the PPV mildly compared with the border/shape term alone, but this increase was of no statistical significance in most cases. On the other hand, combining border/shape terms with a negative finding for “DW hyperintensity” yielded significant changes in PPV, mostly. The following terms showed a statistically significant decrease of PPV when combined with a negative finding for “DW hyperintensity”: circumscribed, non-circumscribed, indistinct, obscured, irregular border, round, oval, lenticular, lobulated, water-drop-shaped, and irregular shape.

**Table 3:** Positive predictive values of border- and shape-related terms describing TZ lesions combined with positive or negative findings for diffusion-weighted hyperintensity. Values are given as PPV in percent (cancer-positive lesions with term(s) marked/all lesions with term(s) marked). PPVs of term combinations were compared with PPVs of single terms using the generalized score test by Leisenring, Alonzo, and Pepe [20]. Differences in PPV were considered significant if *p* values were below 0.05, these values are set in italic. *TZ*, transition zone; *PPV*, positive predictive value; *NPV*, negative predictive value; *DWI*, diffusion-weighted imaging; *TP*, true positives; *POS*, all positives; *TN*, true negatives; *NEG*, all negatives

		Border/shape term alone	Border/shape term combined with positive finding for DW hyperintensity			Border/shape term combined with negative finding for DW hyperintensity		
Feature group	Items	PPV in % (TP/ POS)	PPV in % (TP/ POS)	Relative change compared with border/shape term alone	*p* value	PPV in % (TP/ POS)	Relative Change compared with border/shape term alone	*p* value
Border	Circumscribed	20.4% (22/108)	35.7% (20/56)	+ 15.3%	*0.0013*	3.8% (2/52)	− 16.5%	*0.0016*
	Non-circumscribed	53.3% (16/30)	58.3% (14/24)	+ 5.0%	0.4015	33.3% (2/6)	− 20.0%	*0.0000*
	Indistinct	37.7% (26/69)	43.4% (23/53)	+ 5.7%	0.1838	18.8% (3/16)	− 18.9%	*0.0000*
	Obscured	32.8% (21/64)	41.5% (17/41)	+ 8.7%	*0.0417*	17.4% (4/23)	− 15.4%	*0.0000*
	Irregular	50.0% (30/60)	54.0% (27/50)	+ 4.0%	0.3320	30.0% (3/10)	− 20.0%	*0.0000*
	Spiculated	57.1% (8/14)	57.1% (8/14)	0%	1.0000	- (0/0)	-	NA
	Encapsulated	6.9% (4/58)	16.7% (2/12)	+ 9.8%	0.0000	4.3% (2/46)	− 2.5%	0.3721
	Organized chaos	5.4% (3/56)	12.5% (1/8)	+ 7.1%	0.0000	4.2% (2/48)	− 1.2%	0.5539
	Erased charcoal sign	61.0% (36/59)	60.0% (33/55)	− 1.0%	0.6767	75.0% (3/4)	+ 14.0%	*0.0000*
Shape	Round	21.4% (9/42)	35.0% (7/20)	+ 13.6%	*0.0102*	9.1% (2/22)	− 12.3%	*0.0290*
	Oval	15.6% (12/77)	26.3% (10/38)	+ 10.7%	*0.0031*	5.1% (2/39)	− 10.5%	*0.0068*
	Lenticular	44.7% (17/38)	45.7% (16/35)	+ 1.0%	0.7326	33.3% (1/3)	− 11.4%	*0.0000*
	Lobulated	46.3% (19/41)	52.9% (18/34)	+ 6.6%	0.4198	14.3% (1/7)	− 32.1%	*0.0000*
	Water-drop-shaped	78.6% (11/14)	83.3% (10/12)	+ 4.8%	0.6740	50.0% (1/2)	− 28.6%	*0.0011*
	Wedge-shaped	50.0% (1/2)	50.0% (1/2)	0%	1.0000	- (0/0)	-	NA
	Linear	- (0/0)	- (0/0)	-	NA	- (0/0)	-	NA
	Irregular	39.3% (24/61)	45.7% (21/46)	+ 6.3%	0.1725	20.0% (3/15)	− 19.3%	*0.0000*
DWI	DW hyperintensity	52.0% (105/202)						
	No DW hyperintensity	10.3% (8/78)						

## Discussion

In this study, the predictive power of PI-RADS v2 lexicon terms was analyzed with the aim of adding to the quantitative support of the PI-RADS guideline and identifying areas of improvement.

On the one hand, the presented data corroborates the use of many established assessment criteria in PI-RADS. Lexicon terms indicating a restricted diffusion in PZ lesions showed favorable combinations of both relatively high PPVs and high NPVs (e.g., PPV of 50.7% and NPV of 90.5% for the term “restricted diffusion”). Our work therefore confirms the importance of diffusion-related findings in the PZ [21–23]. Conflicting data have been published regarding the value of DCE imaging in cancer detection. The current consensus is that the addition of DCE imaging to DW imaging increases cancer detection in the PZ, while it might not be useful in TZ lesions [5, 24–27]. Our results support this consensus, with positive DCE findings showing high PPVs up to 68.2% in PZ lesions and poorer performance in TZ lesions with PPV at a maximum of 28.3%. Moreover, signs of invasive behavior or extraprostatic extension are considered highly suggestive of cancer [3]. In accordance with that, our study showed high PPVs for the term “invasion” (76.4% and 58.9% for PZ and TZ lesions, respectively). Features that are suggestive of benign findings, such as “encapsulated” and “organized chaos” for TZ lesions or “linear” for PZ lesions, were consistently associated with benign biopsy outcomes in this study.

On the other hand, our study showed areas of discriminatory potential that are currently not fully utilized in the PI-RADS v2 and v2.1 assessment: In PI-RADS v2, diffusion-related findings play a minor role for TZ scoring [3]. In PI-RADS v2.1, however, the DWI score has gained more importance and scores of 4 and 5 can now upgrade the overall score of a TZ lesion [4]. This adjustment is consistent with studies demonstrating lower ADC values in TZ cancers than in BPH nodules, albeit with a large overlap of ADC values [26, 28–30], and studies showing higher diagnostic accuracy when T2WI- and DWI assessments were combined in TZ lesions [11, 31]**.** The presented data shows another potential of DWI findings: the terms “restricted diffusion,” “diffusion-weighted hyperintensity,” and “ADC hypointense” had high NPVs of 89.4% to 91.8% for TZ lesions, whereas T2WI-related terms showed lower NPVs with a maximum of 66.0% for TZ lesions. Combining T2WI-related border and shape features with a finding of absent diffusion-weighted hyperintensity lowered the respective PPV markedly in most cases compared with the T2WI-related term alone. The means of integrating this negative predictive potential of DWI-related terms into the scoring system in addition to the positive predictive potential of T2WI-related findings could therefore be considered. From the data presented in this study, the absence of features related to restricted diffusion can be assumed to have a good potential to exclude csPCa in the TZ and could be used to downgrade TZ lesions.

Furthermore, we identified a number of border- and shape-related terms with high PPVs, which are currently not explicitly included as criteria for PI-RADS v2 and v2.1 scores: For both PZ and TZ lesions, the descriptors “lenticular,” “lobulated,” and “spiculated” showed rather high PPVs between 44.7 and 58.6%. In TZ lesions, the descriptors “water-drop-shaped,” “irregular,” “non-circumscribed,” and “erased charcoal sign” also showed high PPVs between 50.0 and 78.6%. In a previous study with 14 included patients, Pokharel et al [11] found a similar PPV for TZ lesions with an irregular border (55%). The term “organized chaos” showed a favorable PPV of 5.4% indicating benignity in TZ lesions. Including these highly discriminatory terms into the assessment criteria for PI-RADS categories should be considered.

There are a number of limitations to this study, potentially influencing the generalizability of the results. The standard of reference was histopathologic results of TB and a systematic 10-core biopsy taken ahead of the re-read conducted in this study. Although this method has been shown to have high rates of detection of malignant diseases [32], some malignant lesions may not have been targeted upon TB and may have been missed upon systematic biopsy. Rouvière et al [33] report in a large, prospective, multicenter study that GS ≥ 7 tumor would have been missed in 7.6% (95% CI, 4.6–11.6%) of patients, had TB not been done. The presence of false negatives could influence the reliability of diagnostic values referring to benign-appearing lesions especially, since these were not targeted upon TB. Furthermore, the fact that only patients that underwent biopsy were included may affect the results pertaining to benign-appearing lesions, as patients without suspicious lesions on MRI and low clinical risk factors did not undergo biopsy and were thus excluded from the study. Moreover, benign-appearing lesions in subjects with other suspicious areas may not have been identified as targets by the readers. A more reliable standard of reference would be histopathology after surgical prostatectomy, though this would bias the underlying collective towards medium-aggressive cancers.

Another limitation stems from interreader variability, which was not assessed in this study, as each MRI was read once by a single experienced reader. Additionally, three of the four readers worked at the same institution. The assignment of lexicon terms is, however, a subjective process. Several other studies have shown moderate interobserver agreement for PI-RADS final assessment [8, 34] and few studies have also demonstrated moderate interobserver agreement for assignment of individual lexicon terms [10, 34]. Nevertheless, larger multicenter studies that assess interobserver agreement in the assignment of lexicon descriptors could improve the generalizability of their predictive values.

## Conclusions

The present study identifies lexicon terms with high and low discriminatory power for the prediction of csPCa. The presented data corroborate the importance of DWI/ADC- and DCE-related findings in the PZ by showing favorable PPVs and NPVs in the respective lexicon terms. We identify T2WI-related terms with high PPVs: “markedly hypointense,” “water drop-shaped,” and “spiculated” for the PZ and TZ; “lobulated” for the PZ; and “erased charcoal sign”, “non-circumscribed,” and “irregular border” for the TZ. Moreover, this study demonstrates that DWI/ADC-related lexicon terms can be useful for excluding csPCa in the TZ. We show that combining T2WI-findings with findings of absent DW hyperintensity in TZ lesions significantly decreases PPVs. While with the new PI-RADS v2.1 the positive predictive potential of DWI-findings in TZ lesions has been more prominently utilized, means of incorporating the negative predictive potential of DWI-related terms for TZ lesions (e.g., by downgrading lesions) could further increase diagnostic accuracy.

## Electronic supplementary material

ESM 1 (DOCX 23 kb)

## Abbreviations

AS: Anterior stroma
csPCa: Clinically significant prostate cancer
CZ: Central zone
DCE: Dynamic contrast-enhanced
DWI: Diffusion-weighted imaging
GS: Gleason score
mpMRI: Multiparametric MRI
NPV: Negative predictive value
PCa: Prostate cancer
PI-RADS: Prostate Imaging Reporting and Data System
PPV: Positive predictive value
PZ: Peripheral zone
T2WI: T2-weighted
TB: Targeted MRI/TRUS fusion biopsy
TZ: Transition zone
v2: Version 2
