# Supplementary material for: Validation of the PI-RADS language: predictive values of PI-RADS lexicon descriptors for detection of prostate cancer
Source: Eur Radiol. 2020 Mar 26;30(8):4262–71. doi: 10.1007/s00330-020-06773-1 (PMC7338829; doi:10.1007/s00330-020-06773-1)
Supplement: Supplementary file 1 — (DOCX 23 kb) [file 330_2020_6773_MOESM1_ESM.docx]

***Supplementary Materials***

***Sensitivity and specificity of lexicon terms***

|  |  | **Peripheral Zone** | | **Transition Zone** | |
| --- | --- | --- | --- | --- | --- |
|  | **Terms** | **Sensitivity in %** | **Specificity in %** | **Sensitivity in %** | **Specificity in %** |
| **DWI/ADC features** | Restricted diffusion | 92,0% (104/113) | 46,0% (86/187) | 82,3% (51/62) | 45,3% (63/139) |
|  | DW hyperintensity | 92,9% (105/113) | 48,1% (90/187) | 87,1% (54/62) | 50,4% (70/139) |
|  | ADC hyperintense | 0,0% (0/113) | 98,4% (184/187) | 0,0% (0/62) | 97,8% (136/139) |
|  | ADC isointense | 4,4% (5/113) | 81,8% (153/187) | 6,5% (4/62) | 73,4% (102/139) |
|  | ADC hypointense | 95,6% (108/113) | 22,5% (42/187) | 88,7% (55/62) | 33,8% (47/139) |
| **DCE features*** | Early phase wash-in | 68,6% (48/70) | 65,1% (69/106) | 26,9% (7/26) | 59,7% (46/77) |
|  | Persistent DP (Type 1) | 14,3% (10/70) | 62,3% (66/106) | 26,9% (7/26) | 75,3% (58/77) |
|  | Plateau DP (Type 2) | 38,6% (27/70) | 59,4% (63/106) | 34,6% (9/26) | 66,2% (51/77) |
|  | Washout DP (Type 3) | 42,9% (30/70) | 86,8% (92/106) | 30,8% (8/26) | 70,1% (54/77) |
|  | Positive DCE | 80,0% (56/70) | 57,5% (61/106) | 50,0% (13/26) | 57,1% (44/77) |
|  | Negative DCE | 11,4% (8/70) | 51,9% (55/106) | 50,0% (13/26) | 51,9% (40/77) |
| **T2WI features** | Hyperintense | 2,7% (3/113) | 91,4% (171/187) | 1,6% (1/62) | 77,7% (108/139) |
|  | Isointense | 0,9% (1/113) | 95,7% (179/187) | 1,6% (1/62) | 87,1% (121/139) |
|  | Hypointense | 80,5% (91/113) | 9,1% (17/187) | 82,3% (51/62) | 13,7% (19/139) |
|  | Markedly hypointense | 38,1% (43/113) | 88,8% (166/187) | 33,9% (21/62) | 86,3% (120/139) |
| **Border** | Circumscribed | 48,7% (55/113) | 57,8% (108/187) | 35,5% (22/62) | 38,1% (53/139) |
|  | Non-circumscribed | 12,4% (14/113) | 78,6% (147/187) | 25,8% (16/62) | 89,9% (125/139) |
|  | Indistinct | 32,7% (37/113) | 50,8% (95/187) | 41,9% (26/62) | 69,1% (96/139) |
|  | Obscured | 31,0% (35/113) | 69,0% (129/187) | 33,9% (21/62) | 69,1% (96/139) |
|  | Irregular | 40,7% (46/113) | 68,4% (128/187) | 48,4% (30/62) | 78,4% (109/139) |
|  | Spiculated | 15,0% (17/113) | 93,0% (174/187) | 12,9% (8/62) | 95,7% (133/139) |
|  | Encapsulated | 0,9% (1/113) | 95,7% (179/187) | 6,5% (4/62) | 61,2% (85/139) |
|  | Organized Chaos | 0,9% (1/113) | 96,8% (181/187) | 4,8% (3/62) | 61,9% (86/139) |
|  | Erased charcoal sign | 7,1% (8/113) | 98,9% (185/187) | 58,1% (36/62) | 83,5% (116/139) |
| **Shape** | Round | 12,4% (14/113) | 78,6% (147/187) | 14,5% (9/62) | 76,3% (106/139) |
|  | Oval | 38,1% (43/113) | 72,7% (136/187) | 19,4% (12/62) | 53,2% (74/139) |
|  | Lenticular | 15,0% (17/113) | 93,6% (175/187) | 27,4% (17/62) | 84,9% (118/139) |
|  | Lobulated | 22,1% (25/113) | 89,8% (168/187) | 30,6% (19/62) | 84,2% (117/139) |
|  | Water-drop-shaped | 5,3% (6/113) | 100,0% (187/187) | 17,7% (11/62) | 97,8% (136/139) |
|  | Wedge-shaped | 11,5% (13/113) | 72,2% (135/187) | 1,6% (1/62) | 99,3% (138/139) |
|  | Linear | 0,9% (1/113) | 92,0% (172/187) | 0,0% (0/62) | 100,0% (139/139) |
|  | Irregular | 38,1% (43/113) | 63,6% (119/187) | 38,7% (24/62) | 73,4% (102/139) |
| **Invasion** | Invasion | 37,2% (42/113) | 93,0% (174/187) | 53,2% (33/62) | 83,5% (116/139) |
